# Optimizing Spinal Fusion Cage Design to Improve Bone Substitute Filling on Varying Disc Heights: A 3D Printing Study

**DOI:** 10.3390/bioengineering10111250

**Published:** 2023-10-26

**Authors:** Cheng-Min Shih, Cheng-Hung Lee, Kun-Hui Chen, Chien-Chou Pan, Yu-Chun Yen, Chun-Hsiang Wang, Kuo-Chih Su

**Affiliations:** 1 Department of Orthopedics, Taichung Veterans General Hospital, Taichung 407, Taiwan; 10chengmin@gmail.com (C.-M.S.); 298f@vghtc.gov.tw (C.-H.L.); orthochen@gmail.com (K.-H.C.); adonisvgh@gmail.com (C.-C.P.); 2 Department of Physical Therapy, Hungkuang University, Taichung 433, Taiwan; 3 Department of Post-Baccalaureate Medicine, College of Medicine, National Chung Hsing University, Taichung 402, Taiwan; 4 Department of Rehabilitation Science, Jenteh Junior College of Medicine, Nursing and Management, Miaoli 356, Taiwan; 5 Department of Medical Research, Taichung Veterans General Hospital, Taichung 407, Taiwan; yuchunyen@vghtc.gov.tw (Y.-C.Y.); wangch@vghtc.gov.tw (C.-H.W.); 6 Department of Biomedical Engineering, HungKuang University, Taichung 433, Taiwan; 7 Department of Chemical and Materials Engineering, Tunghai University, Taichung 407, Taiwan

**Keywords:** oblique lumbar interbody fusion, cage, bone graft substitute, 3D printing technology

## Abstract

The success of spinal fusion surgery relies on the precise placement of bone grafts and minimizing scatter. This study aims to optimize cage design and bone substitute filling methods to enhance surgical outcomes. A 3D printed lumbar spine model was utilized to implant 3D printed cages of different heights (8 mm, 10 mm, 12 mm, and 14 mm) filled with BICERA^®^ Bone Graft Substitute mixed with saline. Two filling methods, SG cage (side hole for grafting group, a specially designed innovative cage with side hole, post-implantation filling) and FP cage (finger-packing group, pre-implantation finger packing, traditional cage), were compared based on the weight of the implanted bone substitute. The results showed a significantly higher amount of bone substitute implanted in the SG cage group compared to the FP cage group. The quantity of bone substitute filled in the SG cage group increased with the height of the cage. However, in the FP cage group, no significant difference was observed between the 12 mm and 14 mm subgroups. Utilizing oblique lumbar interbody fusion cages with side holes for bone substitute filling after implantation offers several advantages. It reduces scatter and increases the amount of implanted bone substitute. Additionally, it effectively addresses the challenge of insufficient fusion surface area caused by gaps between the cage and endplates. The use of cages with side holes facilitates greater bone substitute implantation, ultimately enhancing the success of fusion. This study provides valuable insights for future advancements in oblique lumbar interbody fusion cage design, highlighting the effectiveness of using cages with side holes for bone substitute filling after implantation.

## 1. Introduction

Spinal interbody fusion is a surgical procedure commonly employed in the field of orthopedic and neurosurgery. It is typically utilized to treat various spinal conditions, such as degenerative disc disease, spondylolisthesis, spinal stenosis, and spinal deformities like scoliosis. This surgical technique aims to achieve spinal stability and alleviate symptoms by fusing adjacent vertebrae together. Spinal interbody fusion is often per-formed in clinical contexts where conservative treatments have proven ineffective, and patients experience persistent pain, neurological deficits, or spinal instability. The procedure is applicable to a wide range of patients, including those suffering from age-related degenerative conditions, traumatic injuries, or congenital abnormalities affecting the spine. It involves the use of spinal cages made of materials, such as PEEK or titanium alloys, that are implanted into the intervertebral disc space. These cages often have a central void to facilitate bone grafting. Common surgical approaches include posterior lumbar interbody fusion (PLIF), transforaminal lumbar interbody fusion (TLIF), anterior lumbar interbody fusion (ALIF), lateral lumbar interbody fusion (LLIF), and oblique lumbar interbody fusion (OLIF). Retroperitoneal approaches, such as ALIF, OLIF, and LLIF, allow for the placement of larger-footprint spinal cages, which may contribute to increased fusion rates [1,2,3].

The OLIF technique utilizes the natural corridor between the aorta and the psoas muscle, providing direct access to the intervertebral disc space while minimizing the risk of damage to the dural sac, nerve root, posterior muscle and bony structures, thereby reducing surgical trauma and associated complications [4,5]. Furthermore, the OLIF procedure obviates the need for intraoperative neuromonitoring and is relatively straightforward, rendering it a favored surgical approach in recent years [6].

Despite the many benefits of OLIF, there are often challenges encountered during bone-grafting procedures. For instance, when using bone graft or a substitute to fill the cage during the implantation process, the graft may scatter in the retroperitoneal space due to the impact of implantation. Additionally, when using demineralized bone matrix (DBM) to fill the cage, a significant amount of DBM may be scraped off due to insufficient entry height, leading to a decrease in the actual amount of bone graft that is successfully implanted into the intervertebral space and potentially reducing the chances of successful fusion [7]. The main contributing factor to these limitations arises from the current methodology employed in OLIF cage placement, which involves filling the cage with bone graft prior to implantation. However, by designing the cage to be implanted first and subsequently filled with bone graft, these issues can be effectively mitigated.

The application of 3D printing technology in the development of spinal surgery medical devices holds significant promise, particularly in the creation of customized implants tailored to meet individual patient requirements and specific anatomical considerations [8,9,10,11,12,13]. For instance, Fernandes et al. demonstrated, in a previous study, that 3D-printed patient-specific cages offered a larger endplate contact area when compared to commercial off-the-shelf cages [14]. Furthermore, 3D printing technology can enhance the clinical effectiveness of existing medical devices. Adl Amini et al. found, in their prior research, that 3D-printed titanium cages exhibited superior resistance to subsidence following LLIF surgery in comparison to PEEK cages [15]. Additionally, 3D printing technology can be employed in mechanical research pertaining to spinal surgery. For example, Wu et al. utilized finite element analysis to investigate the mechanical performance of 3D-printed cages within the lumbar spine [16].

This study has designed an innovative cage with a side hole that enables the filling of bone substitute after implanting the cage into the disc space, as opposed to the traditional approach where the cage is implanted after placing the bone substitute. The aim of this study is to utilize 3D printing technology to fabricate cage and spinal models, allowing for a comparative evaluation of the two different cage designs and the measurement of bone substitute retention within the cage at varying disc heights.

## 2. Materials and Methods

To assess the efficacy of intervertebral fusion surgery and compare different methods of filling bone substitute materials during cage implantation, this study primarily utilized 3D printing to fabricate the required surgical instruments and simulate the cage-implantation process. Therefore, in this study, models of the spine and cages (one of them is a specially designed innovative cage) were constructed. Furthermore, to accommodate the varying intervertebral disc heights encountered in clinical practice, 3D printing was employed to create cages of different heights, thus allowing for the simulation of potential clinical scenarios.

### 2.1. Model of Spine

**Figure 1:**
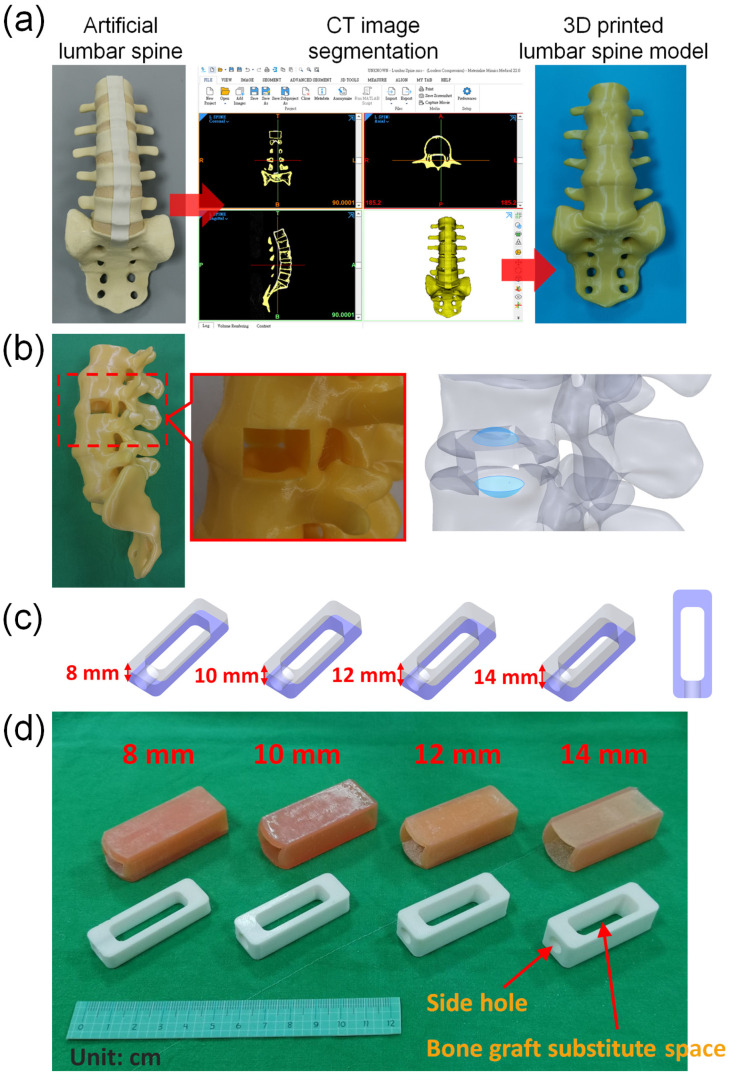
(**a**) A lumbar spine model was created using 3D-printing technology. (**b**) The model included two concave spaces on the lower endplate of L3 and the upper endplate of L4, which simulated the gap that exists between the cage and endplates after implantation of the cage in the disc space during a clinical procedure. (**c**) Cages with identical cross-sectional areas but varying heights were used in this study. (**d**) 3D-printed cage model and disc model used in this study.

The lumbar spine model used in this study is the computed tomography (CT) image obtained by tomographic scanning using the commercially available artificial lumbar spine model (SKU:1352, Pacific Research Laboratories, Inc., Vashon, Washington, DC, USASawbones Inc., USA), and then the CT image is imported into the medical image segmentation software Mimics (Mimics Medical 22.0, Materialise, Leuven, Belgium) to reconstruct the spine model (Figure 1a). Only the bones and intervertebral discs are selected during image selection. After removing ligaments and other irrelevant tissues, a computer model of the spine and intervertebral discs is established. Afterwards, the selected vertebral model was used to create a 16 mm × 22 mm depth 50 mm space on the left side of the intervertebral disc between the vertebrae L3 and L4 using three-dimensional computer-aided design (CAD) software (Solidworks 2021, Dassault Systemes SolidWorks Corp, Waltham, MA, USA). In addition, two concave spaces were established on the lower endplate of L3 and the upper endplate of L4 to simulate the gap between the cage and the endplates after the cage is implanted in the disc space in clinical practice. After the computer model of the lumbar spine is established and saved as an STL file, the lumbar spine model STL is imported into a 3D printer (PING DUAL300, Linkin factory Co., Ltd., Taichung, Taiwan) for physical model printing. The printing resolution of this 3D printer is a nozzle diameter of 0.4 mm and a layer height of 0.2 mm. The drawn lumbar spine model is saved as an STL file and imported into the software PING Slicer2.1 for slicing. The material is yellow PLA (Poly Lactic Acid) material (the wire diameter of the material is 1.75 mm), and the setting of the printer when printing is a layer height of 0.2 mm, fill density of 15%, print speed of 40 mm/s, printing temperature of 220 °C, and bed temperature of 60 °C. The 3D printing model of the spine for this study has been printed (Figure 1b).

### 2.2. Model of Cage

This study aimed to evaluate the effects of different cage heights. The cage used in this simulation measured 50 mm in length and 18 mm in width, and included a rectangular space that measured 34 mm in length and 11 mm in width, with heights of 8 mm, 10 mm, 12 mm, and 14 mm, respectively. Due to the fixed length and width of the space within the cage, the capacity for bone grafting within the cage increases proportionally with different heights. The respective volumes are 2.93 cm^3^, 3.662 cm^3^, 4.395 cm^3^, and 5.128 cm^3^ (Figure 1c). Additionally, a circular hole with a diameter of 7.5 mm was created in the center of one of the short sides of the cage. After creating four computer models of the cage with different heights, the cage models were saved as STL files and imported into the software PING Slicer2.1 for slicing. The material used was white PLA (Poly Lactic Acid) with a wire diameter of 1.75 mm. The printing machine was set to a layer height of 0.2 mm, a fill density of 100%, a print speed of 40 mm/s, and a printing temperature of 220 °C. The base plate temperature was set to 60 °C (Figure 1d).

### 2.3. Model of Disc

**Figure 2:**
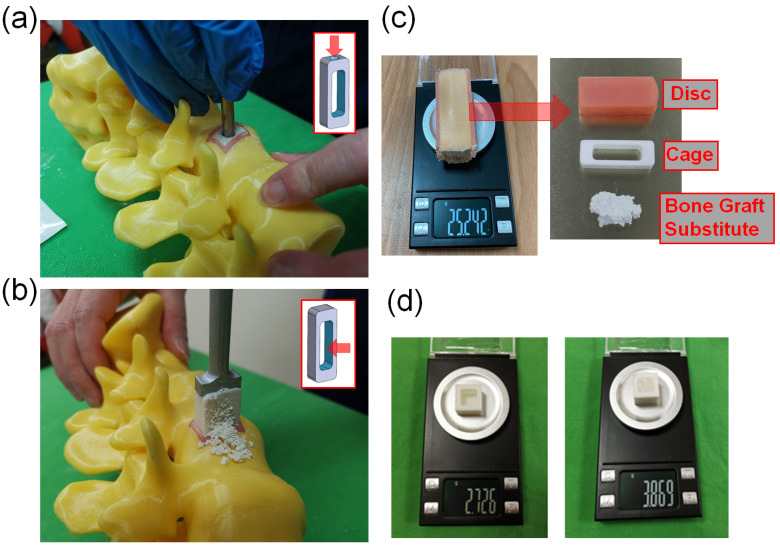
(**a**) The SG cage group implanted the cage first, and then filled it with bone substitute through the holes on the cage. (**b**) The FP cage group packed bone substitute into the interior space of the cage using the finger-packing technique. (**c**) The intervertebral disc substitute, cage, and bone substitute were removed together and weighed using a high-precision scale. (**d**) The weight of bone substitute filled by finger packing in a standard 1 cubic centimeter space.

In addition, in order to evaluate the use of different heights of cage implantation, this study used soft 3D printing materials to simulate the site where cage was implanted in the intervertebral disc. Therefore, SolidWorks software was used to create computer models that match different cage heights and are suitable for insertion design. The size of the space designed on the spine model is 16 mm × 22 mm, and the depth is 50 mm, and a computer model of the intervertebral disc is established to simulate the implantation site for 4 different cage heights. Afterwards, the computer model created was printed with a 3D printer (Phrozen Sonic Mini 4K, Phrozen tech co., Hsinchu, Taiwan). The STL file of the computer model was imported into the slice software CHITUBOX 64 for slice. The material of the simulated intervertebral disc is MSLA Spring Resin Pink. The print parameters were layer height 0.1 mm, bottom layer count 6, transition layer count 6, transition type: linear, exposure time 6 s, bottom exposure time 30 s, light-off delay 15 s, bottom light-off delay 15 s, bottom lift distance 8 mm, lift distance 8 mm, bottom lift speed 80 mm/min, lift speed 60 mm/min, and retract speed 120 mm/min (Figure 2).

### 2.4. Preparation of Bone Substitute

Synthetic calcium phosphates are commonly utilized as bone substitutes in clinical practice. In this study, BICERA^®^ Bone Graft Substitute, which comprises 60% hydroxyapatite (HA) and 40% calcium phosphate (TCP), with a pore size of approximately 461.92 ± 90.66 μm [17], was used. Normal saline, an FDA-approved solution commonly used for fluid resuscitation in human patients, was used to mix with bone substitute at a ratio of 2 g of normal saline per 5 g of BICERA^®^ Bone Graft Substitute, to simulate the real surgical procedure of filling bone substitutes into the cage.

### 2.5. Experimental Methods

1. A model with an internal volume of one cubic centimeter was created and weighed using a high-precision balance. The prepared bone substitute was then filled into the cubic centimeter using the finger-packing technique and weighed again to determine the density of the bone substitute after finger packing.
2. The cages produced by 3D printing were placed into soft material-printed intervertebral disc simulators of corresponding height, and their total weight was measured using a high-precision balance. It was observed that the cages with heights of 8 mm, 10 mm, 12 mm, and 14 mm had total weights of 17.901 g, 16.779 g, 16.070 g, and 15.535 g, respectively.
3. In this study, we used two methods to place bone substitute into cages. The SG cage group (side hole for grafting group) involved using a cage with a side hole to fill it with bone substitute after implantation (Figure 2a). The FP cage group (finger-packing group) involved filling the cage with bone substitute before implantation using finger packing (Figure 2b).
  a. SG cage group: This study employed a cage with filling side holes (a specially designed innovative cage with “side hole for grafting”). Firstly, a soft-material printed intervertebral disc was placed into a 3D-printed spinal model, followed by the implantation of the specially designed cage into the soft-material printed intervertebral disc. Bone substitute was then filled into the cage through the side holes until it was completely filled. Subsequently, the intervertebral disc substitute, cage, and bone substitute were removed together and weighed using a high-precision scale (Figure 2c). This step was repeated 10 times for each group based on the different heights of the cage.
  b. FP cage group: Traditional methods were used in this study. Firstly, a soft 3D-printed intervertebral disc was placed into a 3D-printed spine model. Next, bone substitute was packed into the interior space of the 3D-printed cage using the finger-packing technique. An inserter was then employed to connect the 3D-printed cage, and a hammer was used to aid the implantation of the 3D-printed cage into the soft intervertebral disc within the spine model. After implantation, the weight of the 3D-printed intervertebral disc, cage, and internal bone substitute was measured using a high-precision scale and recorded. This step was repeated 10 times for each group based on the height of the cage.
  c. After each use of the high-precision scale, it must be recalibrated to zero before subsequent measurements can be taken.
4. A 3D-printed cube with a 1-cubic centimeter internal space was fabricated for measurement purposes. The weight of the empty 3D-printed cube was initially determined. Subsequently, the 1-cubic centimeter space within the cube was filled with the bone substitute material, and the total weight was measured. This enabled the calculation of the weight of the bone substitute when occupying a volume of 1 cubic centimeter, which was found to be approximately 1.143 g (Figure 2d).

### 2.6. Statistical Methods

In the SG cage group, as each set of data represents an independent event, and the independent events exhibit a normal distribution with homogeneity of variance, an ANOVA test was performed. If the null hypothesis was rejected, an LSD post-hoc test was used to determine the mean value comparisons between each subgroup. Significant differences (*p* < 0.5) were observed between each subgroup using ANOVA and LSD post hoc tests.

In the FP cage group, as the grouped data were all independent events exhibiting a normal distribution, but not homogeneity of variance, a Welch’s test was used to determine if the mean values across all groups were equal. If the null hypothesis was rejected, a Dunnett T3 post-hoc test was used for pairwise comparisons.

We compared the two bone substitute filling methods separately for each cage height, and used an independent sample *t*-test if the data for the two subgroups met the criteria of independent events, normal distribution, and homogeneity of variance.

## 3. Results

This study analyzed the weight of bone substitute implanted using two different filling methods, and the results showed that the SG cage group had a significantly higher amount of bone substitute.

**Figure 3:**
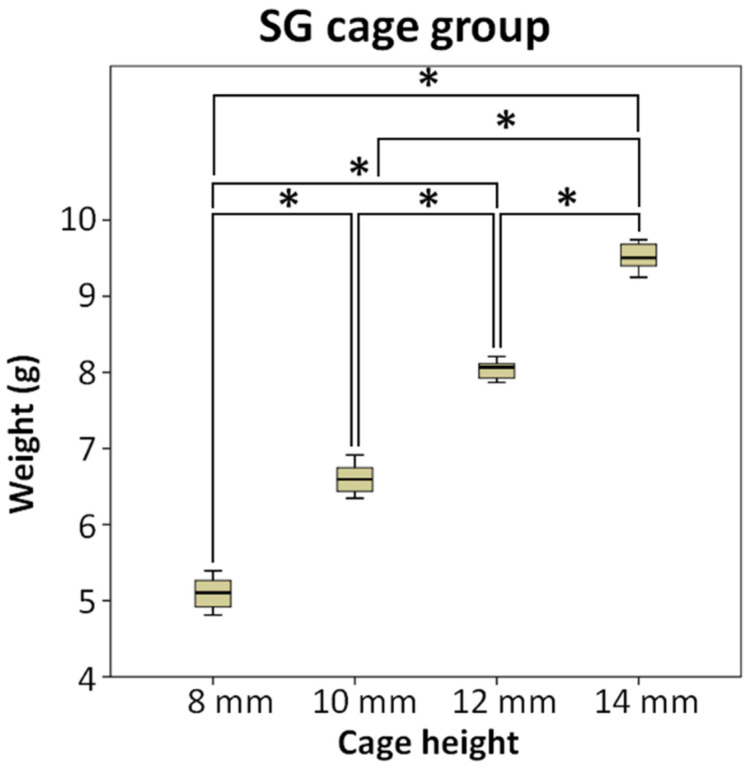
The SG cage group compared the weight of bone substitute implanted after using cages of different heights, * means significant at *p* < 0.5.

Figure 3 demonstrates that the amount of bone substitute filled in the SG cage group follows the order of cage heights: 8 mm (5.10 g), 10 mm (6.60 g), 12 mm (8.04 g), and 14 mm (9.52 g), respectively. As the cage height increases, the available space for filling the bone substitute also increases proportionally, resulting in a higher weight of bone substitute that can be accommodated. Using ANOVA and LSD post-hoc tests, significant differences (*p* < 0.5) were observed among each subgroup.

**Figure 4:**
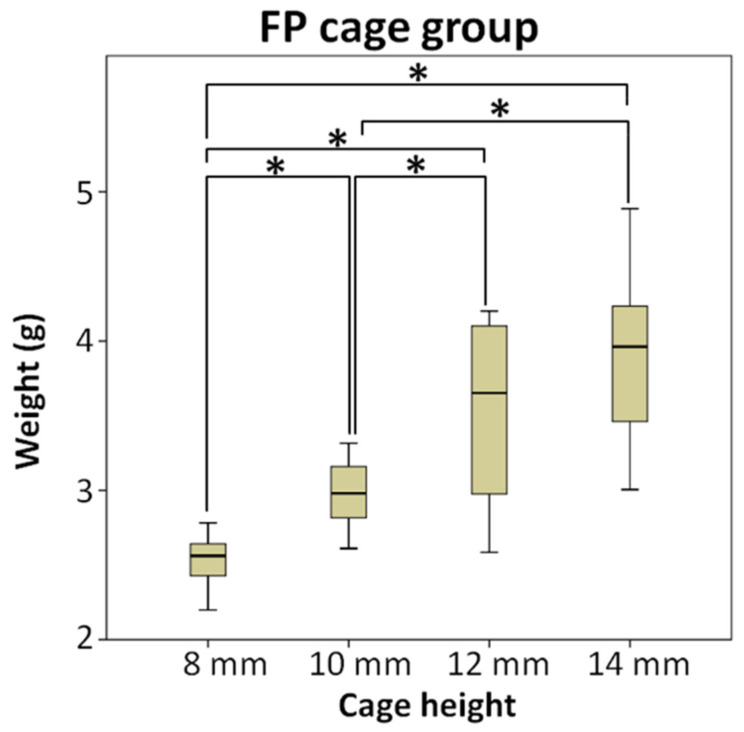
The FP cage group compared the weight of bone substitute implanted after using cages of different heights, and the test results indicated no significant difference between the two subgroups of 12 mm and 14 mm, * means significant at *p* < 0.5.

Figure 4 shows the conditions of different height cages and bone grafts implanted in the cage in the FP cage group. In the FP cage group, the weight of the measured bone substitute sequentially increased to 2.54 g, 2.98 g, 3.54 g, and 3.93 g as the cage height increased. The test results indicated no significant difference between the two subgroups of 12 mm and 14 mm.

**Figure 5:**
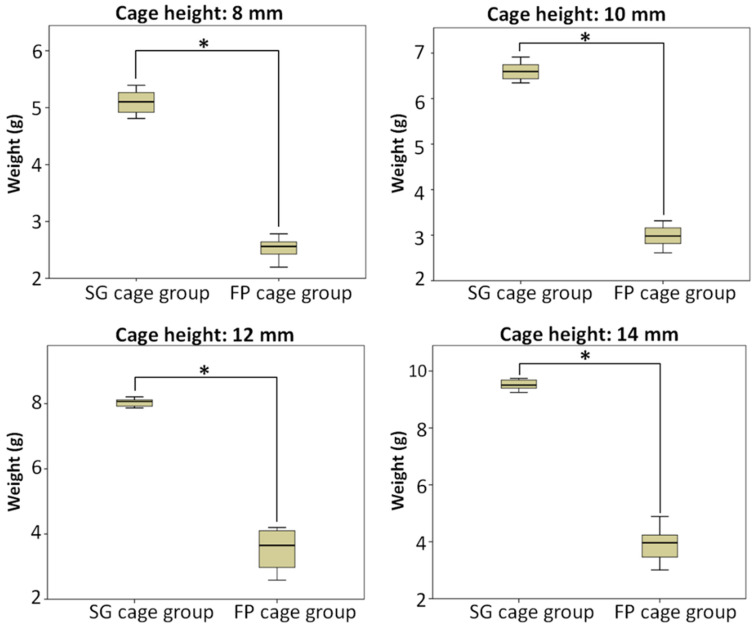
Compared the weight of bone substitute implanted using two different bone-grafting techniques when cages of the same height were implanted, and the SG cage group being greater than FP cage group (*p* < 0.5) and statistically significant in all subgroups, * means significant at *p* < 0.5.

Figure 5 compares two different methods for implanting bone grafts into the cage: the SG cage group and the FP cage group. The results are displayed in separate charts based on four different heights. Additionally, the study compared the means and quartiles of the two bone-grafting methods between cages at the same height, grouped according to different cage heights. Independent sample *t*-tests showed significant differences in bone substitute weights between the two grafting methods in all groups. The SG cage group exhibited significantly greater weights than the FP cage group (*p* < 0.5), indicating statistical significance.

The weight of a standard 1 cubic centimeter space filled with finger-packing bone substitute was measured to be 1.143 g. Based on the volume available for bone grafting in cages of different heights, the estimated weights after filling with bone substitute were 3.349 g, 4.187 g, 5.024 g, and 5.861 g, respectively. The SG cage group had significantly higher bone graft weights in each cage height compared to their estimated weights, with weight percentages of 152%, 158%, 160%, and 162% in order of cage height. In contrast, the FP cage group had significantly lower bone graft weights than their estimated weights, with weight percentages of 76%, 71%, 71%, and 67% in order of cage height (Table 1).

## 4. Discussion

In this study, we investigated two different methods of filling the cage with bone substitute during spinal interbody fusion. We found that inserting the cage into the disc space first and then using a bone graft side hole to fill the cage resulted in a significant increase in the volume of bone substitute filling the disc space.

Our experiment showed that in the SG cage group, the higher the cage height, the greater the amount of implanted bone substitute, and a linear relationship was observed between the amount of bone substitute and the filling space inside the cage. For cages with the same height, recording the volume of bone grafting ten times resulted in a smaller standard deviation, indicating stable bone-grafting volume. In the FP cage group, similar to the SG cage group, the trend of the amount of bone substitute increasing with the increase of cage height was observed. However, in the data of the 12 mm and 14 mm subgroups, the Welch’s test and Dunnett T3 post hoc test (*p* < 0.5) showed no significant difference, which may be due to the inconsistent amount of bone substitute scattered outside the cage during the process of hammering the insertor to implant the cage, resulting in insignificant differences in the measured bone graft weight in these two subgroups. In addition, in the FP cage group, the recorded amount of bone substitute for each subgroup had a larger standard deviation in the measured weight, indicating that the bone substitute was scattered outside the cage, making it difficult to stably control the amount of bone grafting each time.

**Figure 6:**
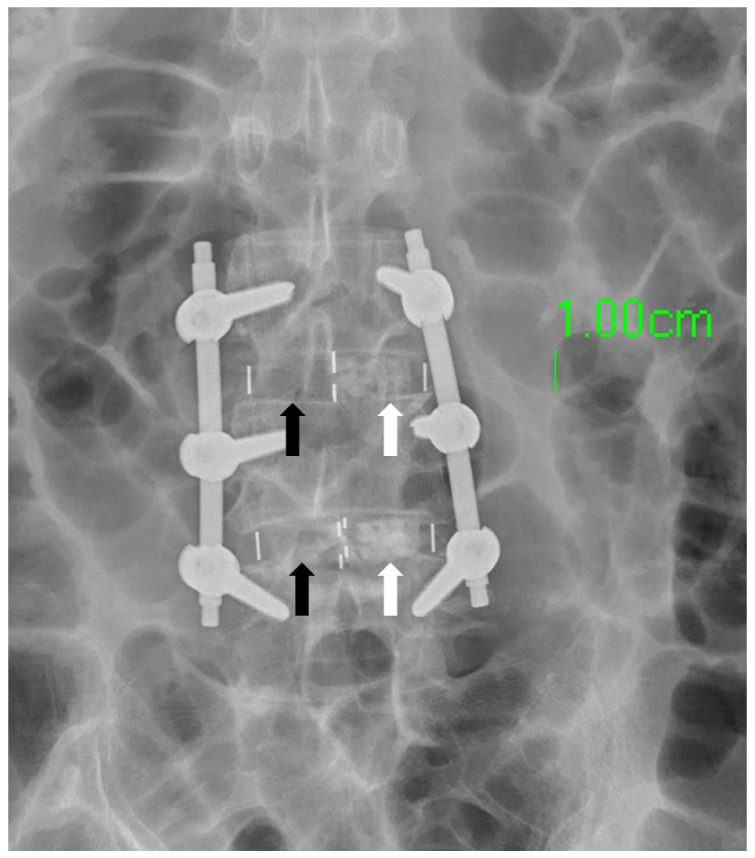
The white arrow designates the localization of the bone substitute, whereas the black arrow signifies its conspicuous absence within the designated region. This absence is attributed to the inadvertent scattering of the bone substitute into the retroperitoneal cavity during the cage-implantation procedure.

**Figure 7:**
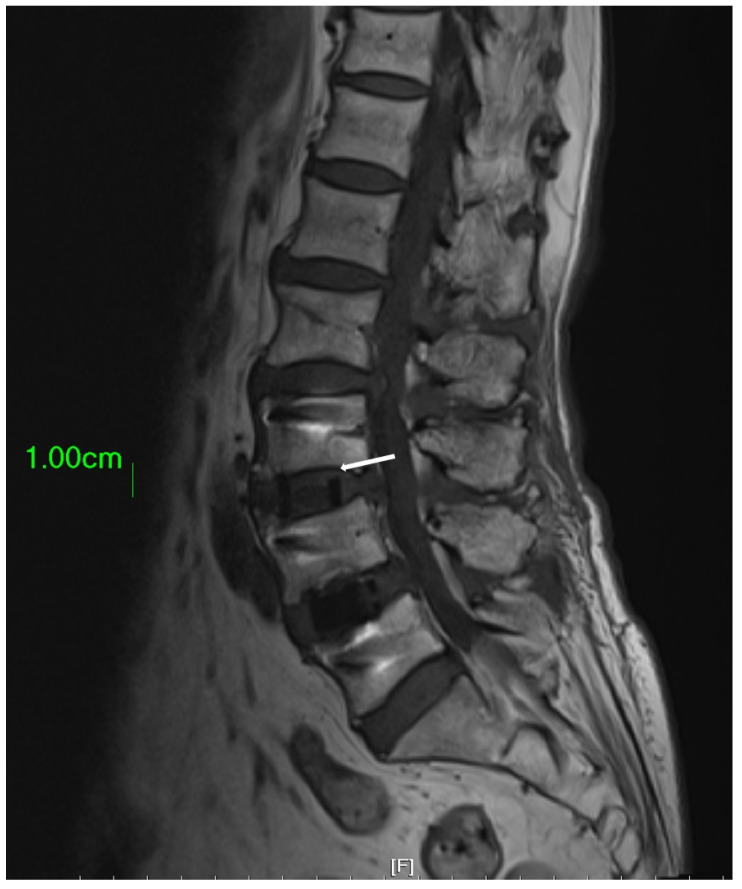
The area highlighted by the white arrow demonstrates a gap that exists between the endplate and the cage. In circumstances where the cage is filled with bone substitute material prior to its implantation into the disc space, this gap cannot be adequately filled by the bone substitute.

In clinical practice, the amount of bone graft used can have an impact on the success of fusion surgery. Insufficient bone graft volume may lead to fusion failure [18]. The amount of bone graft can be observed using X-Ray imaging [19]. The OLIF cage offers a substantial volume for surgeons to fill with bone substitute. However, the conventional finger-packing technique, followed by hammering the inserter for cage implantation, can lead to bone substitute dispersal into the retroperitoneal space, resulting in insufficient bone substitute retention within the cage (Figure 6). Such a significant reduction in the cage’s bone substitute filling may potentially decrease the success rate of subsequent interbody fusion. Therefore, this study employed a cage with bone graft side holes. The cage was initially placed into the disc space, and bone substitute was subsequently packed through specially designed side holes, effectively increasing the volume of bone graft genuinely implanted within the disc space, with the aim of enhancing the fusion success rate. Additionally, there is a gap between the upper and lower sides of the cage center and the endplate of the intervertebral disc, which cannot be filled with bone graft using the conventional method of implanting the cage [20,21]. Due to the presence of this gap, the vertebral body may have difficulties integrating with the cage, ultimately leading to nonunion. However, if the cage is first implanted into the disc space and then filled with bone graft through the side holes, this gap can be effectively filled (Figure 7).

This study has several limitations. Firstly, the rationale for employing 3D printing technology was the absence of commercially available cage designs featuring the SG cage group configuration in the clinical market at the time of the study. Consequently, we manufactured these specialized cages ourselves to facilitate comparative research and assess their performance in various bone-grafting methods. In this regard, 3D printing technology served the specific research objectives, enabling a comparison of different implants and methods. Furthermore, the decision to use 3D-printed cages instead of clinically available ones allowed for customization of their production to suit the specific research needs, providing greater control over minimizing differences between the two cage groups and avoiding discrepancies arising from distinct geometric shapes. It should be noted that the actual cages are shuttle-shaped, whereas, in this experiment, rectangular-shaped 3D-printed cages were employed, closely approximating a rectangular design. The choice of a rectangular design was primarily driven by the focus on investigating bone substitute implantation. Shuttle-shaped cages are more irregular and prone to bone substitute dis-lodgment; hence, the rectangular design was selected. Additionally, the rectangular design facilitated volume calculations in this study and bone graft filling, reducing operational differences between groups. Secondly, when constructing the spinal model, it would have been ideal to design appropriately sized soft intervertebral disc holes directly to better simulate the clinical scenario. However, to accommodate the measurement of implanted bone substitute weight, the simulated intervertebral disc portion was made removable to facilitate the weight measurement of bone substitutes. Thirdly, in actual surgery, surgeons often mix bone substitutes with blood. However, in this study, normal saline was used instead of blood. This decision aimed to standardize the quality of the bone substitute by mixing it with normal saline in a fixed proportion. Normal saline is a commonly used solution for fluid resuscitation in human patients and is FDA-approved. These specific limitations were intentionally designed as part of the experimental methodology to minimize differences resulting from these factors. Additionally, we conducted in vitro experiments in this study to evaluate the impact of cage geometry on post-implantation outcomes. Therefore, the material properties of the cage had minimal influence within the scope of this research. Consequently, we employed commonly used 3D printing material, PLA, for evaluation. It is important to note that in current clinical practice, cages are primarily constructed using materials such as titanium or PEEK [22,23,24]. Therefore, for the future clinical adoption of the cage design proposed in this study, it would be imperative to use materials that are biocompatible for direct implantation into the human body.

The main objective of this research is to validate two different techniques for implanting bone substitutes within cages of different heights using 3D printing. There have been many previous studies that used 3D-printed models to conduct research and evaluation on the spine [25,26]. This study utilized 3D-printed materials with consistent properties as substitutes for the lumbar spine. Additionally, the study aimed to simulate the clinical scenario of a surgeon inserting the cage, providing a realistic tactile experience. Therefore, 3D printing was employed to create the spine models for experimentation. Furthermore, by employing 3D printing, we can quickly and customarily optimize implant design. Furthermore, in addition to simulating and evaluating medical devices through 3D printing [27,28,29,30,31,32], future investigations may incorporate finite element analysis for mechanical simulations [33,34,35]. In addition, future research can use commonly used clinical materials (titanium or PEEK) to make cage models, and then conduct cadaver study, sawbones study, clinical study, or biomechanical evaluation of other implant designs. Through the validation of our 3D-printed models, this study provides valuable reference information on bone grafting and surgical techniques for medical device manufacturers and clinical practitioners. By enabling greater volumes of bone grafting, it may enhance the fusion success rates, thus benefiting patients [18].

## 5. Conclusions

The OLIF cage with side holes enables the sequential implantation of the cage followed by bone substitute filling, effectively reducing the issue of bone substitute scattering outside the cage during implantation. Moreover, it can address the problem of insufficient fusion surface area caused by gaps between the cage and endplates that cannot be effectively filled with bone graft. In addition, this design also allows a cage with a larger Bone graft substitute space to be filled with more bone grafts. This study aims to provide a reference for future improvements in OLIF cage design, especially with the use of the innovative cage design introduced in this research, which incorporates side holes to facilitate an increase in the volume of bone graft substitute that can be filled into the cage.

## Figures and Tables

**Table 1:** The data of estimated weights after filling with bone substitute, average weight of SG cage group, and average weight of FP cage group.

Cage Height		8 mm	10 mm	12 mm	14 mm
volume (cm^3^)	1	2.930	3.662	4.395	5.128
Estimated weight (g)	1.143	3.349	4.187	5.024	5.861
Average weight (g) of SG cage group ± SD (average weight/estimated weight)		5.101 ± 2.003 (152%)	6.603 ± 0.190 (158%)	8.040 ± 0.118 (160%)	9.516 ± 0.169 (162%)
Average weight (g) of FP cage group ± SD (average weight/excepted weight)		2.540 ± 0.175 (76%)	2.980 ± 0.224 (71%)	3.535 ± 0.585 (71%)	3.928 ± 0.552 (67%)

## Data Availability

Not applicable.
